# Acrofacial vitiligo secondary to PI3KCA inhibitor, alpelisib: case report

**DOI:** 10.3389/fonc.2023.1255832

**Published:** 2023-10-30

**Authors:** Maayan Geller Hinich, Adham Hijab

**Affiliations:** ^1^ Oncology Division, Ziv Medical Centre, Safed, Israel; ^2^ Radiotherapy Unit, Ziv Medical Centre, Safed, Israel

**Keywords:** vitiligo, alpelisib, breast cancer, PIK3CA, skin adverse effects

## Abstract

Alpelisib plus fulvestrant is a valid second or advanced line of treatment for patients with metastatic hormone receptor (HR)-positive, HER2-negative breast cancer who harbor an activating PIK3CA mutation. The well-known side effects of alpelisib are hyperglycemia, rash, and diarrhea. Herein, we report a case of a woman who developed diffuse depigmented macules on the face, arms and legs, three months after initiating alpelisib. Both clinical and histopathological findings were consistent with new-onset vitiligo. To our knowledge, this is the first case described in literature which suggests a causal relationship between alpelisib and irreversible dermatological adverse effect.

## Introduction

Around 40% of patients with hormone receptor (HR)- positive, HER2 negative breast cancer have an underlying activating mutation in PIK3CA gene which encodes the catalytic p110α subunit of the phosphatidylinositol 3-kinase (PI3K) class I enzyme (1). Alpelisib is an α-selective PI3K (PIK3CA) inhibitor which is taken orally. The pivotal SOLAR-1 trial, demonstrated a clinical benefit of alpelisib plus fulvestrant in patients with HR-positive, HER2-negative, advanced breast cancer with PIK3CA mutation, who progressed on previous hormonal therapy (2). Indeed, based on these results, alpelisib plus fulvestrant has gained FDA approval and is an established second or subsequent line of treatment for patients with advanced hormonal-positive, HER2-negative breast cancer with a PIK3CA mutation. The most frequently encountered adverse events that are associated with alpelisib are hyperglycemia, gastrointestinal symptoms (diarrhea, nausea), and rash.

Vitiligo, is an irreversible depigmentation disorder, that is occasionally observed as a consequence of treatment with checkpoint inhibitors. Various tyrosine kinase inhibitors could also induce pigmentary changes including hypopigmentation and hyperpigmentation. To date, there are no reports of vitiligo secondary to PIK3CA inhibitor, alpelisib. We present a case of a woman who developed vitiligo few months after starting this novel targeted agent.

## Patient information

A 46 years old, otherwise healthy, premenopausal woman was diagnosed in 2016 with regionally advanced infiltrating ductal carcinoma (stage IIIA T3N1M0) of right breast. The tumor was positive for hormone receptors’ expression (ER and PR positive), without HER2 amplification (HER2 + 1 by immunohistochemistry), and with a Ki67 between 5% to 10%. She went on to receive neoadjuvant chemotherapy (with a documented minimal response), followed by mastectomy with axillary node dissection, and adjuvant radiotherapy. Residual tumor was still evident in the right breast (scattered foci of grade 3 invasive ductal carcinoma, same hormonal and HER2 profile) with 7 out of 12 dissected axillary lymph nodes involved by carcinoma. Adjuvant hormonal therapy with tamoxifen was initiated along with ovarian suppression.

In 2021, patient developed recurrence (biopsy-proven) with a disseminated disease in liver, mediastinum, and lungs. Hormonal and HER2 profile was consistent with the initial tumor diagnosed in 2016. Treatment with CDK4/6 inhibitor (Ribociclib) plus fulvestrant was commenced. In June 2022, a disease progression in liver and bones was documented. Molecular profiling revealed PIK3CA mutation and treatment with alpelisib plus fulvestrant was started in August 2022. Shortly after, patient developed hyperglycemia which required dose reduction of alpelisib (dose reduced from 300 mg to 250 mg daily).

## Clinical findings

**Figure 1:**
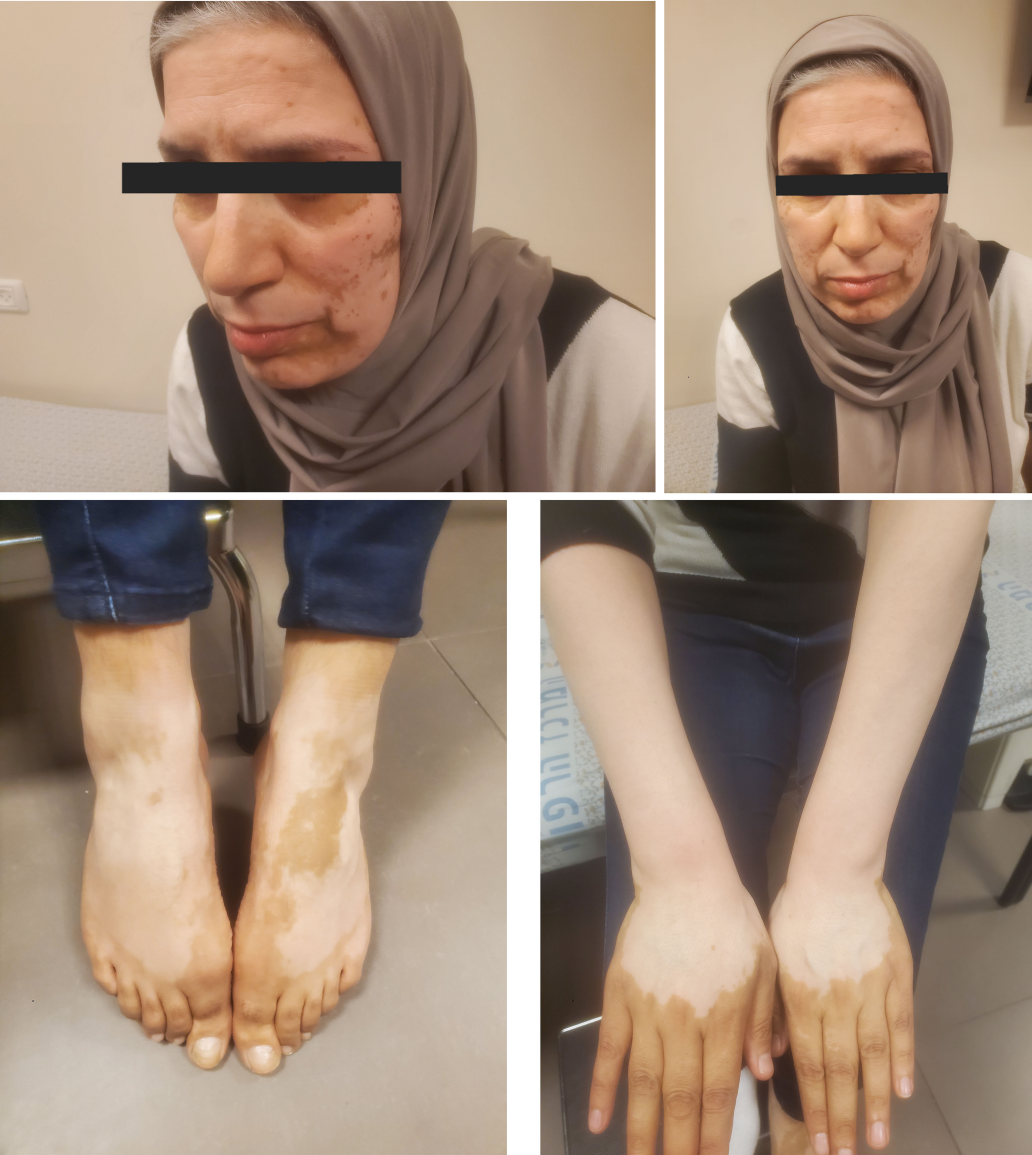
Diffuse areas of depigmented skin apparent in the areas of face, arms, and lower legs.

Three months after the initiation of alpelisib, patient developed skin discoloration in arms, legs, and face that was very suggestive of vitiligo (**Figure 1**). She was referred to a comprehensive dermatologic assessment. A skin biopsy was obtained, which was remarkable for atrophic epidermis with scattered melanophages in the upper dermis. Notably, the patient did not have neither personal or familial history of dermatological disorders nor did not start any new concomitant medications. Soon after this, the patient experienced a substantial disease progression in the form of visceral crisis due to significant deranged liver function tests, she went on to receive doublet chemotherapy comprised of carboplatin and gemcitabine.

## Discussion

Although not life-threatening, vitiligo could profoundly impact quality of life as a result of a significant change in appearance. In the medical oncology field, vitiligo is commonly known to be associated with immunotherapy. In this regard, sometimes patients find comfort in the fact that it could be a positive predictive biomarker for treatment response (3). An autoimmune mechanism is one of the main hypothesis behind the development of vitiligo, therefore the association between checkpoint inhibitors and vitiligo is tangible. However, melanocytes destruction caused by oxidative stress secondary to radical oxygen species (ROS), is another plausible mechanism of vitiligo (4). The Nuclear factor erythroid 2-related factor2 (Nrf2) is a downstream component of the PI3K/Akt pathway and is a major transcription factor of ROS scavengers (5, 6). In addition, the expression of anti-apoptotic proteins such as BCL-2 is upregulated, while pro-apoptotic molecules such as caspase 3 and 9 is attenuated by the activation of PI3K/Akt pathway (7), thus preventing apoptotic death in melanocytes which could be secondary to oxidative stress. Therefore, inhibition of PI3KCA by alpelisib could hamper melanocytes’ ability to manage oxidative stress leading to melanocytes’ destruction and eventually to vitiligo.

Despite its rarity, both clinicians and patients alike should be aware of the chances of developing this sequela as it is generally irreversible and could be quite disfiguring, leading to psychological distress and anxiety.

## Data availability statement

The original contributions presented in the study are included in the article/supplementary material. Further inquiries can be directed to the corresponding author.

## Ethics statement

Written informed consent was obtained from the individual(s) for the publication of any potentially identifiable images or data included in this article.

## Author contributions

AH: Writing – original draft, Writing – review & editing. MH: Writing – original draft.
